# Modified-release hydrocortisone (Efmody^®^) in children with congenital adrenal hyperplasia: a retrospective registry study

**DOI:** 10.3389/fendo.2026.1854737

**Published:** 2026-07-07

**Authors:** Erwin Lankes, Levin Wiebelt, Kathrin Bettina Helge, Dirk Schnabel, Peter Kühnen, Oliver Blankenstein, Uta Neumann

**Affiliations:** 1 Department of Pediatric Endocrinology and Diabetology, Charité - Universitätsmedizin Berlin, ENDO-ERN Center, Berlin, Germany; 2 Center for Chronic Sick Children, Charité - Universitätsmedizin Berlin, Berlin, Germany; 3 Institute of Biometry and Clinical Epidemiology, Charité – Universitätsmedizin Berlin, Berlin, Germany; 4 German Center for Child and Adolescent Health (DZKJ), Partner Site Berlin, Berlin, Germany

**Keywords:** 17-hydroxyprogesterone, children, congenital adrenal hyperplasia, disease-control, growth, hydrocortisone modified-release capsules, treatment

## Abstract

**Background:**

Replacement therapy for congenital adrenal hyperplasia (CAH) in childhood includes hydrocortisone and, in salt-wasting forms, addition of fludrocortisone. Immediate-release hydrocortisone often fails to adequately suppress early morning 17-hydroxyprogesterone (17OHP) levels. Hydrocortisone modified-release capsules (HMRC, Efmody^®^) were approved by the EMA in 2021 for the treatment of patients aged ≥12 years with CAH. This study evaluates the efficacy and safety of HMRC in children and adolescents based on real-world data collected from the I-CAH registry.

**Methods:**

A single-center retrospective analysis of children with CAH was conducted. Linear mixed models (LMMs) were used to calculate pre–post comparisons of 17OHP saliva profiles, growth rate, bone age, body mass index, hydrocortisone and fludrocortisone dosage, blood pressure, and safety parameters before and during HMRC therapy (study registration, https://drks.de/search/de, DRKS00038319).

**Findings:**

The median age of the 36 children with CAH at the time of treatment switching to HMRC was 10 years (interquartile range [IQR]: 8–14 years). Of these, 15 were prepubertal and 21 were (post)pubertal. HMRC therapy was associated with a deceleration of previously increased growth velocity (pre-switch: 0.1 SDS/year, 95% CI: 0.0 to 0.2; post-switch: -0.1 SDS/year, 95% CI: –0.2 to 0.1) and with a reduction of median difference of bone age and chronological age by 0.50 years (IQR -1.17–0.27, range -2.2–2.00). Mean hydrocortisone dose increased by 2.4 mg/m²/day during HMRC treatment. Mean morning 17OHP concentrations significantly decreased from 337 ng/L (95% CI: 240–437) to 214 ng/L (95% CI: 155–294) after switching to HMRC treatment. No adrenal crisis was observed.

**Interpretation:**

Twice-daily HMRC therapy can also improve CAH therapy in children and adolescents in terms of hormonal control and growth.

## Introduction

Congenital adrenal hyperplasia (CAH) is a rare genetic disorder most commonly caused by steroid 21-hydroxylase deficiency, resulting in cortisol deficiency and, in severe cases, concomitant aldosterone deficiency. Impaired cortisol synthesis diminishes negative feedback on the hypothalamic–pituitary–adrenal (HPA) axis, leading to elevated adrenal production of 17-hydroxyprogesterone (17OHP) and androgens (1). Therapy includes hormone replacement with hydrocortisone (HC) and, if necessary, with fludrocortisone (FC). Due to its shorter half-life and better titratability, immediate-release hydrocortisone is recommended for growing children, while long-acting, high-potency glucocorticoids such as dexamethasone are not recommended in clinical guidelines (2). In addition, the dose should not exceed 15–17 mg/sqm body surface area (BSA) in children and adolescents, as it has been shown to have a negative effect on adult height (3, 4). Hydrocortisone therapy is intended to replace missing cortisol, mimicking the daily cortisol rhythm and thereby suppressing increased ACTH and androgen production, along with the associated effects on growth, metabolism, and other endocrine systems (5). This leads to a recommended dosage of immediate-release hydrocortisone given three to four times daily. Avoiding the early morning ACTH surge is crucial and can be achieved by early morning dosing or inverse dose distribution with equal or higher evening doses (6). In recent years, a modified-release hydrocortisone in capsules (HMRC) was developed to address the nocturnal rise of ACTH and thereby improve the therapy of CAH (7–9). The multi-particulate formulation consists of a microcrystalline core coated with hydrocortisone and a polymer coat, which causes the delayed drug release not until pH >6.8, which physiologically occurs in the last third of the small bowel. Nevertheless, twice-daily dosing is required to mimic physiological cortisol secretion, with the highest dose at bedtime to achieve early morning peak levels (8). HMRC is available in 5 and 10 mg capsules, authorized by the EMA in May 2021 and marketed in Germany since September 2021 for adults and children ≥12 years. Clinical data in children are lacking, but pharmacokinetic models indicate similar pharmacokinetic behavior in adolescents (10).

This study will report the results of a single center’s experience of HMRC treatment of children and adolescents since September 2021 based on real-world data collected from the I-CAH registry, with a particular focus on biochemical disease control, children’s growth, safety, and hydrocortisone dosage.

## Methods

### Study design

This is a single-center retrospective, observational study in children and adolescents with CAH due to 21-hydroxylase deficiency in a real-world setting to analyze a pre–post comparison of patients whose treatment was switched from immediate-release hydrocortisone to HMRC since September 2021. If the children were under 12 years of age at the start of HMRC treatment, the child and parents were informed and advised on the off-label treatment. Patients were included in the analysis if at least two measurements of each, auxological parameters and a daily 17OHP profile, were available before and after the treatment switch. We excluded patients from the analysis if the children were suffering from non-classic CAH, receiving treatment other than immediate-release hydrocortisone or had no hydrocortisone treatment before switching to HMRC, and if the patients were without 21-hydroxylase deficiency. Treatment switch was defined as switching evening administration or all administrations to HRMC. We hypothesize that switching treatment will lead to an improvement in biochemical disease control, measured as a reduction in 17OHP levels.

### Data review

Data were collected with the I-CAH registry (platform of I-SDM registries, https://sdmregistries.org/), an international registry platform authorized by the National Research Ethics Service in the United Kingdom as a research database containing information gathered during routine clinical care (11). Participation in the registry and analysis of the data have been approved by the local ethics committee (EA2/007/24), and data were entered into the registry after obtaining informed consent from patients and their guardians. The study is registered in the German Clinical Trials register (DRKS00038319). The following anthropometric and treatment data from the longitudinal module of the I-CAH registry were used: height, body mass index (BMI), body surface area, systolic and diastolic blood pressure (sys-BP, dias-BP), genital stage and breast stage, bone age, parental height, and hydrocortisone and fludrocortisone doses. The anthropometric data were measured, usually every 3 months, at a clinical appointment in the pediatric endocrinology department. Blood pressure was measured using the RIVA-ROCCI (RR) method (12). Continuous time was calculated relative to the treatment switch (in years or months). A total of 36 patients were included in the analysis set, with a median of 35 available observations of body height per patient (IQR 27–42) from birth to treatment switch and nine (7–11) from treatment switch to February 2025 (the database’s closing date). Height, BMI, sys-BP, and dias-BP measurements were transformed to z-scores using Robert-Koch-Institute (RKI) percentiles of a German healthy reference cohort (13). Measurements up to 3 years after birth were excluded from analysis due to the lack of reference percentiles. Bone age measurements before and after the treatment switch were available for 13 of the 36 patients, which were included in the analysis. Bone age was determined using the method of Greulich and Pyle (14). If more than one examination for a child was available during the pre- or post-switch phase, the mean of the test values was included in the analysis.

To monitor the quality of hydrocortisone treatment, salivary 17OHP was measured (Salivette^®^, Sarstedt, Nümbrecht, Germany) ideally every 3 months. Samples are collected at home in the morning, afternoon, and evening just before taking HC medication on two consecutive days and conveyed by postal service. HC dosing was adjusted based on 17OHP levels (6, 15). For analysis, 17OHP values up to 2.25 years before the switch and up to 3 years after the switch were available. After switching to HMRC, afternoon samples were not collected regularly and therefore excluded from analysis. Categories are defined differently for morning (4:00 a.m. to 10:59 a.m.) and evening (17:00 p.m. to 3:59 a.m.). In the morning, 0–75 is considered “overtreatment”, 75–200 is considered “good disease control”, 200–300 is “acceptable”, and >300 ng/L is considered “undertreatment”. For evening measurements, the ranges were reduced as follows: 0–25 is considered “overtreatment”, 25–75 is “good disease control”, 75–200 is “acceptable”, and >200 ng/L is considered “undertreatment”. 17OHP measurements were censored by a lower limit of quantification (LLOQ) of 3.6 and by an upper limit of quantification (ULOQ) of 1,000. Four samples with values lower than LLOQ were replaced with 2.5 (3.6/sqrt(2)), and 47 samples with values larger than ULOQ were replaced with 1,414.2 (1,000*sqrt(2)). Replaced values were used for data analysis.

Pubertal status is measured according to Tanner (16) as breast stage (female) and genital stage (male), with stage 1 corresponding to prepubertal status, 2 and 3 to pubertal status, and 4 and 5 to postpubertal status.

### Statistical analysis

To calculate the average period effect of treatment switch on 17OHP, we used linear mixed models (LMMs) with fixed period effect, random intercept by patient, and random intercept for measurements on subsequent days. Reported confidence intervals (CI) were calculated on the log-scale and back-transformed with bias correction. The *p*-value of the fixed period effect is reported. Additionally, we calculate and report separately for morning- and evening-17OHP the percentage of time spent in the range for good or overtreatment for each patient separately before and after switching to HMRC. Time intervals with missing information are dropped from time-in-range calculation. For descriptive analyses (swimmer plot, violin plot), the 17OHP values of two consecutive days were averaged, whereas for modeling-based analyses (pre-/post-averages, *p*-values), two values entered the model, while the model was adjusted for the correlation of repeated within-patient measurements.

LMMs were used to assess pre–post changes in outcome trajectories before and after the switch to HMRC therapy, with endpoints including height (“growth”), BMI, sys-BP, and dias-BP. We specify fixed linear and quadratic time effect for the pre-period and a fixed linear interaction effect of time and period. As random effects, we specify a random linear time effect, random linear time–period interaction, and random intercept with unstructured covariance matrix. Marginal change rates and confidence intervals are calculated by least squares for the two periods, respectively (17), with reference time point being treatment switch (time = 0). The shift in change rate between pre- and post-period is assessed by a test on time–period interaction (18). The quadratic fixed time effect for the pre-switch period is added due to the large number of measurements before treatment switch and is favored over a model with only linear time effects by the Akaike information criterion (AIC).

*Post-hoc* subgroup analysis for the endpoint body height is reported for prepubertal children at switch versus patients being pubertal or postpubertal at switch.

For body height, an additional graphical analysis is reported. The *z*-scores are centered by parental target height using RKI percentiles for age 18 and Hermanussen’s formula (19). Centered *z*-scores of body height measure the difference to parental target height in reference cohort standard deviations. Patient-period averages are computed with an LMM with fixed period effect and random intercept and period effect by patient. Random effects for each patient and period correspond to the average difference from parental target height in the respective period and are visualized by violin plots.

Hydrocortisone (mg/sqm/day) dosages are reported descriptively by visit across patients using violin plots. The last available dosage and BSA were used to calculate relative hydrocortisone dosages. In case of two visits of a subject within one visit interval, the last visit was displayed. Missing visits at the start or end of the observation period were not imputed. Missing visits between two observed visits (intermediary missing) were imputed by last observation carried forward (LOCF) if dosage is known for these intermediary missing visits. To calculate the average dosage changes for hydrocortisone as well as fludrocortisone, LMMs with random intercept by patient are used, where analysis is restricted to an interval of 3 years before and after treatment switch.

All reported *p*-values should be interpreted as explorative and are not adjusted for multiple testing. All calculations were performed using R [v. 4.1.1] (20) and the packages dplyr [v. 1.1.4] (21), lubridate [v. 1.9.3] (22), emmeans [v.1.10.6] (17), and merTools [v. 0.6.2] (23). Graphs were created using ggplot2 [v. 3.5.1] (24).

## Results

**Figure 1:**
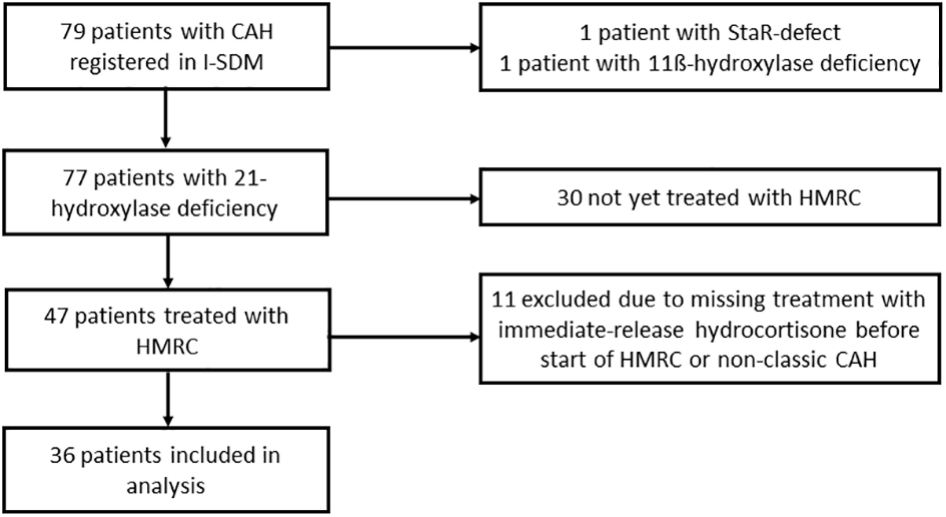
Flowchart of patients with CAH registered in I-SDM registry from Charité Universitätsmedizin Berlin and included in the study.

A total of 79 patients from our clinic are registered with the I-SDM registry for a CAH diagnosis. Of these patients, 36 patients with CAH (19 male) fulfilled the inclusion criteria and were included in the HMRC treatment analysis since September 2021 (**Figure 1**).

At the start of the HMRC treatment, the median age was 10 years (IQR 8–13, range 5–22), 15 children were prepubertal (Tanner 1), and 21 patients were pubertal or postpubertal (Tanner 2–5). Although auxological data were available for all children, the values for 17OHP were only available and analyzed for *n* = 31 (morning) and *n* = 29 (evening). Since some patients have started the HMRC treatment only recently, the number of measurements in the post-switch period is limited in these cases. All 36 patients included in the analysis had classic CAH and were treated with HC and FC (**Table 1**).

**Table 1:** Sample characteristics for each analysis set.

Group	Total (n = 36)	Biochemical analysis (n = 31)
Male	19 (53%)	17 (55%)
Age at switch (range)	10 (5, 22)	11 (5, 22)
Pubertal status at switch		
Tanner 1	15 (42%)	14 (45%)
Tanner 2-5	21 (58%)	17 (55%)

### Biochemical disease control

**Figure 2:**
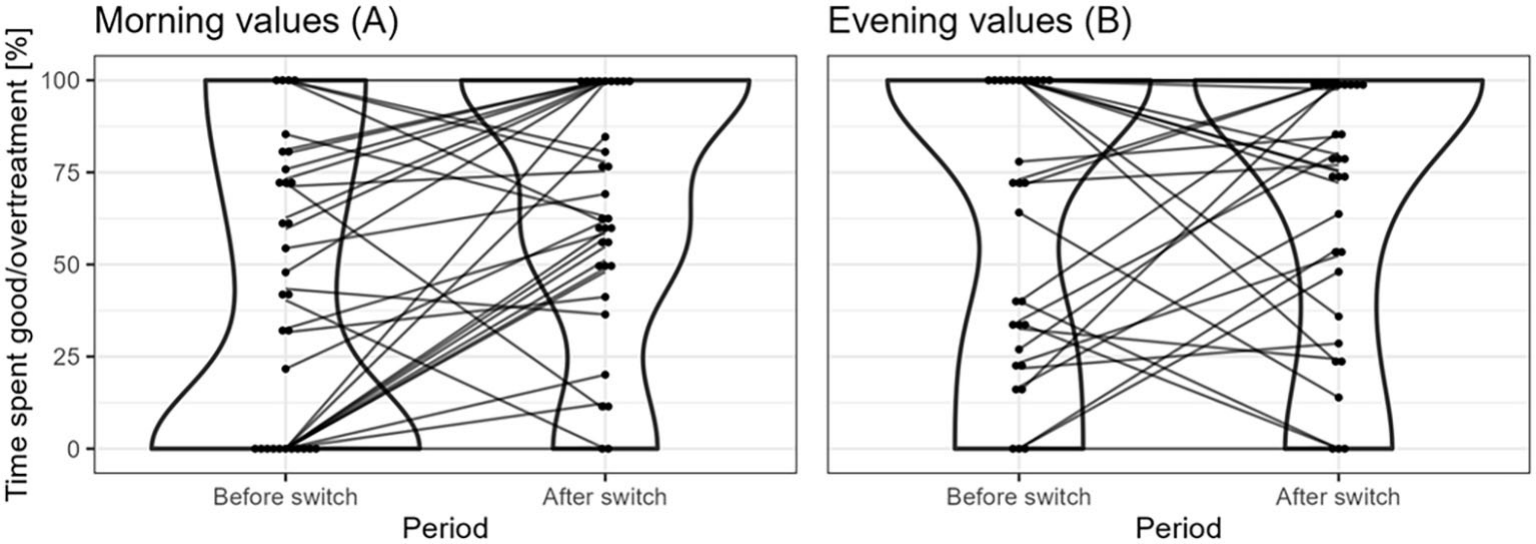
This violin plot shows the percentage of time spent in the 17-OHP range for good or overtreatment of the disease, in the morning (**(A)**/left) and evening (**(B)**/right), before and after switching to HMRC. Time intervals with missing information are dropped from time-in-range calculation. Number of patients analyzed: *n* = 31 for morning values and *n* = 29 for evening values. For morning values and evening values, respectively, each patient is represented by two dots, where one is the value before treatment and one is after treatment start with HMRC. The values of a patient are connected by a line.

**Figure 3:**
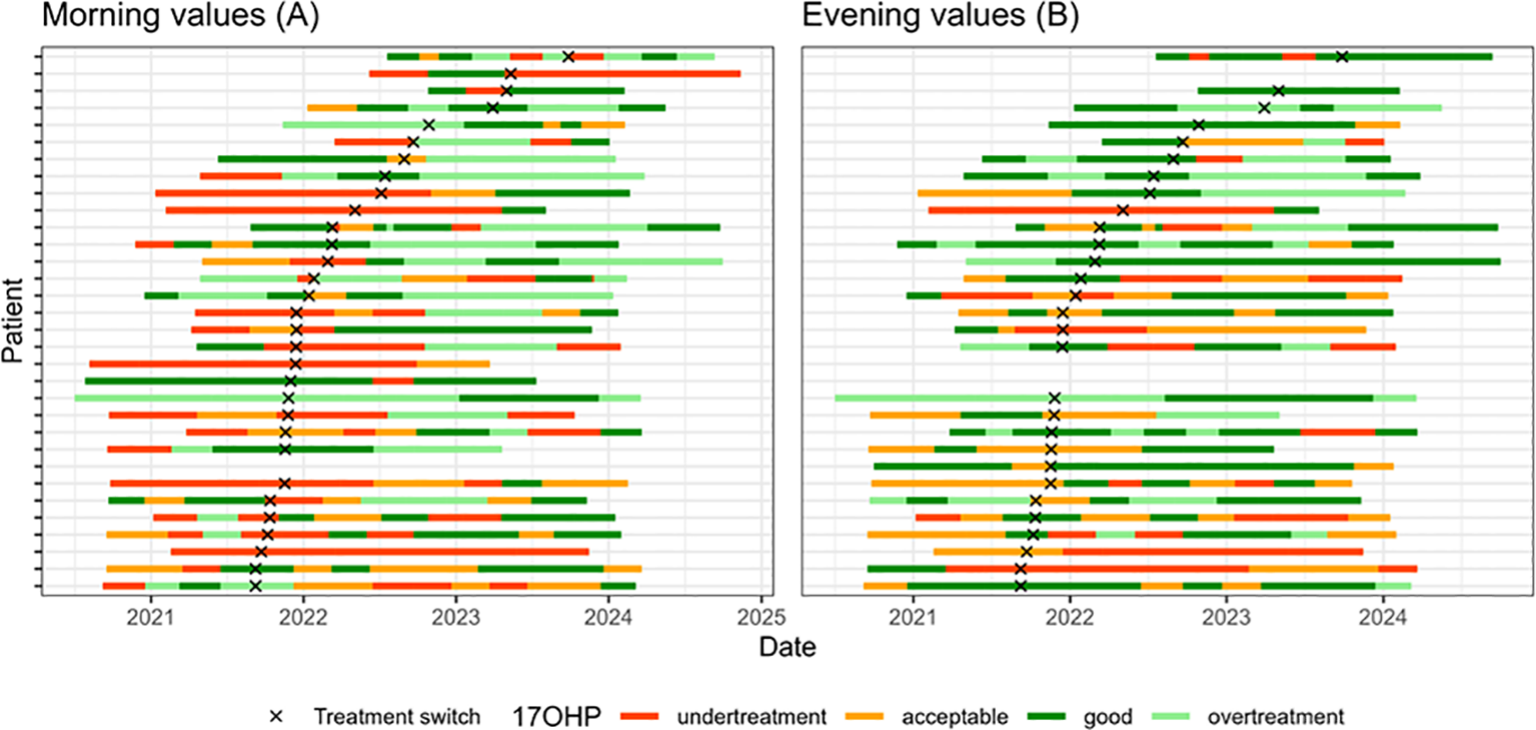
Classification of disease control (undertreatment—red, acceptable—orange, good—dark green, and overtreatment—light green) according to 17OHP values in the morning **(A)** and in the evening **(B)** before administration. Each horizontal line corresponds to one patient. Number of patients analyzed: *n* = 31 for morning values and *n* = 29 for evening values.

The morning 17OHP-values in the post-switch period were, on average, reduced compared to those of the pre-switch period by a factor of 0.64 (*p* < 0.01), with average levels before the switch at 337 ng/L (95% CI 240–473 ng/L) and after the switch at 214 ng/L (95% CI 155–294 ng/L) (**Table 2**). This corresponds to disease control that was classified as undertreatment prior to HMRC treatment and showed acceptable average control during treatment. **Figure 2** shows the individual course of disease control assessed by time-on-study spent in good or overtreated disease control according to saliva sampling before and during HMRC treatment. Before the HMRC treatment started, 11 of the 31 children (35%) were assigned undertreated based on their morning values at any time, while in 14 children (45%) therapy control was predominantly good or showed overtreatment (at least 50% of the individual time on study). Four children had good 17OHP values or showed overtreatment in all available measurements. Following the switch to HMRC, 24 children (77%) had predominantly good or overtreated disease control. In nine children (29%), the 17OHP sample every morning showed good or overtreated disease control. In 11 children (35%), therapy control has improved during HRMC treatment, showing predominantly good or overtreated disease control. Only one child experienced deterioration in therapeutic control below the threshold of 50% time on study after the therapy change. In six children, the quality of control remained poor both before and after the change and in two of them at any time. The evening 17OHP levels were not significantly different during the pre- and post-switch periods (**Figures 2**, **3**).

**Table 2:** Outcome parameter estimates for each period.

Outcome parameter	Before treatment switch to HMRC	After treatment switch to HMRC	*p*-value	Sample size		
	Estimate (95% CI)	Estimate (95% CI)		Number of patients	Number of measurements	
Avg. morning 17OHP (ng/L)	337 (240, 473)	214 (155, 294)	< 0.01	31	474	
Avg. evening 17OHP (ng/L)	91 (66, 125)	103 (77, 139)	0.24	29	341	
Avg. **change** in body height (z-scores/year)	0.1 (0.0, 0.2)	-0.1 (-0.2, 0.1)	0.02	36	1,615	
Avg. BMI (z-scores)	0.6 (0.2, 0.9)	0.6 (0.1, 1.2)	/	36	1,595	
Avg. **change** in BMI (z-scores/year)	0.0 (-0.1, 0.0)	0.0 (-0.1, 0.1)	0.36	36	1,595	
Avg. **level** in sys-BP (z-scores/year)	0.3 (0.2, 0.4)	0.2 (0.0, 0.3)	0.42	36	1,222	
Avg. **level** in dias-BP (z-scores/year)	0.2 (0.1, 0.3)	0.0 (-0.1, 0.2)	0.80	36	1,222
Avg. HC-dose (mg/sqm/day)	12.4 (11.6, 13.2)	14.8 (14, 15.6)	/	36	657
Avg. FC-dose (µg/day)	75.8 (64.4, 87.2)	87.5 (76.1, 98.9)	/	36	657

Linear mixed model parameters by study period. Each row corresponds to a separate model. Hydrocortisone dose is given in mg/meter square (sqm) body surface area (BSA) and analyzed for patients with classic CAH and fludrocortisone dose in µg/day. For height- and BMI-trend, the *p*-value reported is calculated from a time–period interaction test within an linear mixed model (LMM), where period main effect is omitted on purpose. For 17OHP-endpoints, *p*-values are calculated from a test of main period effect. Point estimates are calculated as least squares, while confidence intervals are calculated as predictions over a reference grid. For trend analyses (height, BMI), the reference timepoint for reported estimates is treatment switch. *P*-values should be interpreted as exploratory and are not adjusted for multiple testing.

Avg., average; BMI, body mass index; sys-BP, systolic blood pressure; dias-BP, diastolic blood pressure; HC, hydrocortisone; FC, fludrocortisone; 17OHP, 17-hydroxyprogesterone.

### Height

**Figure 4:**
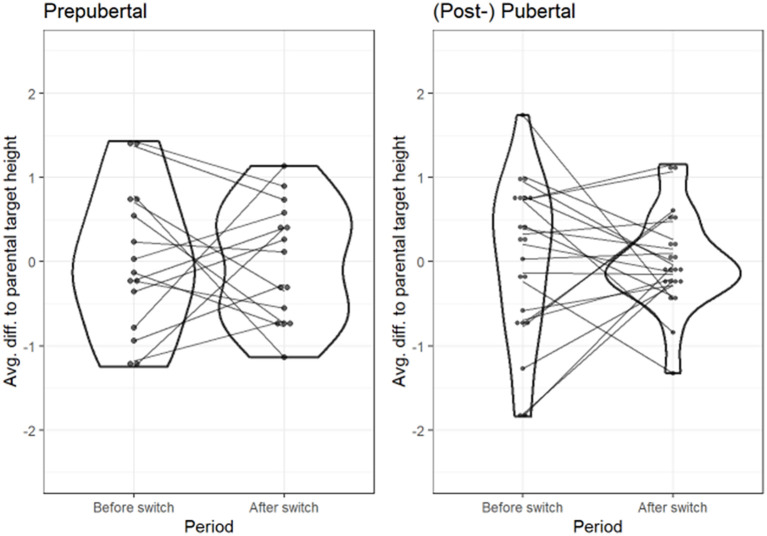
Average difference to parental target height in z-scores by patient, period, and subgroup. Average difference is calculated as the random intercept of a LMM with fix period and subgroup and interaction effect and random patient effect. Number of patients analyzed: *n* = 36. Each patient is represented by two dots, where one is the value before treatment switch and one is after treatment switch. The values of a patient are connected by a line.

In the following, 95% CIs are displayed in “[]”. Average differences from parental target height are reduced on average. The distribution after treatment switch is more concentrated at values around 0 and approached parental target height better after treatment switch (**Figure 4**).

**Figure 5:**
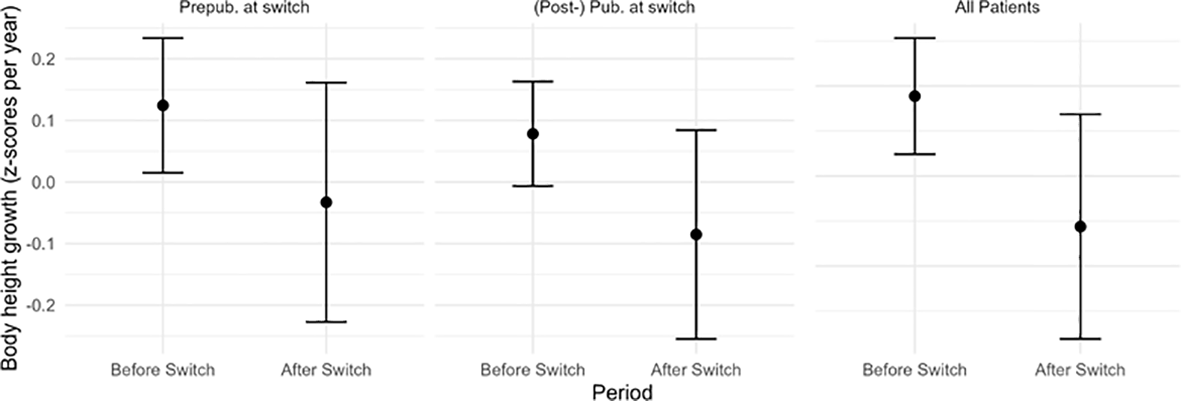
Confidence intervals for change in body height for full analysis set and two subgroups. In each pair, left CI corresponds to pre-switch period and right CI to post-switch period. Interaction test between subgroup and period yields *p* = 0.80. A total of 36 patients and 1,615 measurements were used for analysis. “All” column is calculated as reported for models without subgroups. Subgroup models “prepubertal” and “postpubertal and pubertal” are calculated within one model, where fixed and random effect structure are as for the model without subgroups but the time–period interaction term is dropped and a time–period–subgroup interaction term is added to the model.

We observed, on average, growth rates higher than in the healthy reference cohort before treatment switch (0.1 [0.0 to 0.2] *z*-scores per year) and growth rates lower than the reference after the switch (-0.1 [-0.2 to 0.1] *z*-scores per year). The test for interaction of period (pre vs. post) and the pubertal development at treatment switch prepubertal (Tanner 1) vs. postpubertal (Tanner 2–5) yielded *p* = 0.80 (1,615 measurements in 36 patients) (**Table 3**). An average decrease in growth was observed in both subgroups, for postpubertal patients (before treatment switch: 0.1 [0.0 to 0.2] *z*-scores per year, after switching to HMRC: -0.1 [-0.3 to 0.1] *z*-scores per year) compared to prepubertal children at switch (before treatment switch: 0.1 [0.0 to 0.2] *z*-scores per year, after switching to HMRC: 0.0 [-0.2 to 0.2] *z*-scores per year) (**Table 3**, **Figure 5**).

**Table 3:** Subgroup analysis of body height growth.

Average change in body height (z-scores/year)	Before treatment switch to HMRC	After treatment switch to HMRC	*p*-value	Sample size	
	Estimate (95% CI)	Estimate (95% CI)		Number of patients	Number of measurements
Pubertal development at switch			0.80	36	1,615
Patient prepubertal at switch	0.1 (0.0, 0.2)	0.0 (-0.2, 0.2)			
Patient (post)pubertal at switch	0.1 (0.0, 0.2)	-0.1 (-0.3, 0.1)			

### Bone age

**Figure 6:**
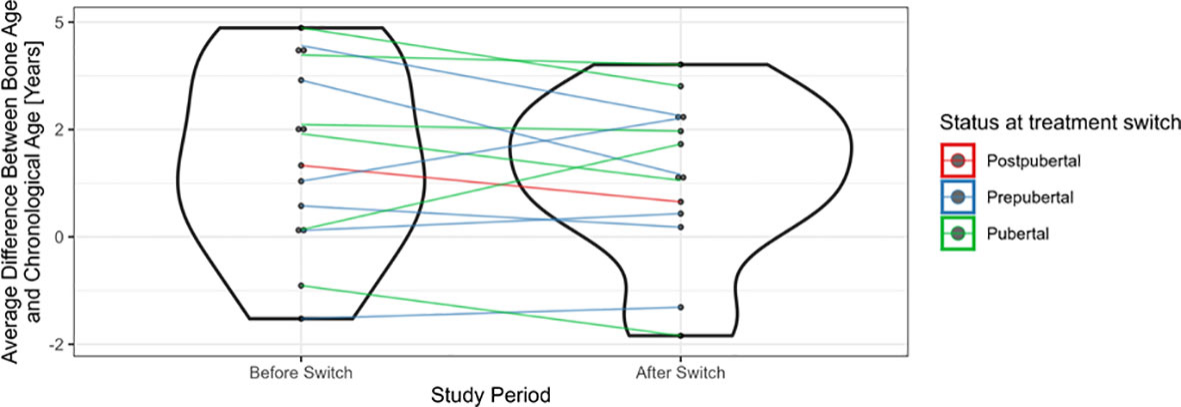
Average difference between bone age and chronological age in years. Number of patients analyzed: *n* = 13. Each patient is represented by two dots, where one is the value before treatment switch and one is after treatment switch. The values of a patient are connected by a line. The color of the line indicates pubertal status at treatment switch (green—prepubertal, blue—pubertal, and red—postpubertal).

The analysis of bone age included data from 13 children for whom bone age measurements were available both before and after treatment switch (female, *n* = 6). Following the switch to HMRC treatment, there was a convergence between bone age and chronological age and, consequently, a reduction in acceleration (**Figure 6**). The median difference of bone age and chronological age decreased by 0.50 years (IQR -1.17–0.27, range -2.2–2.00).

### Body mass index

**Figure 7:**
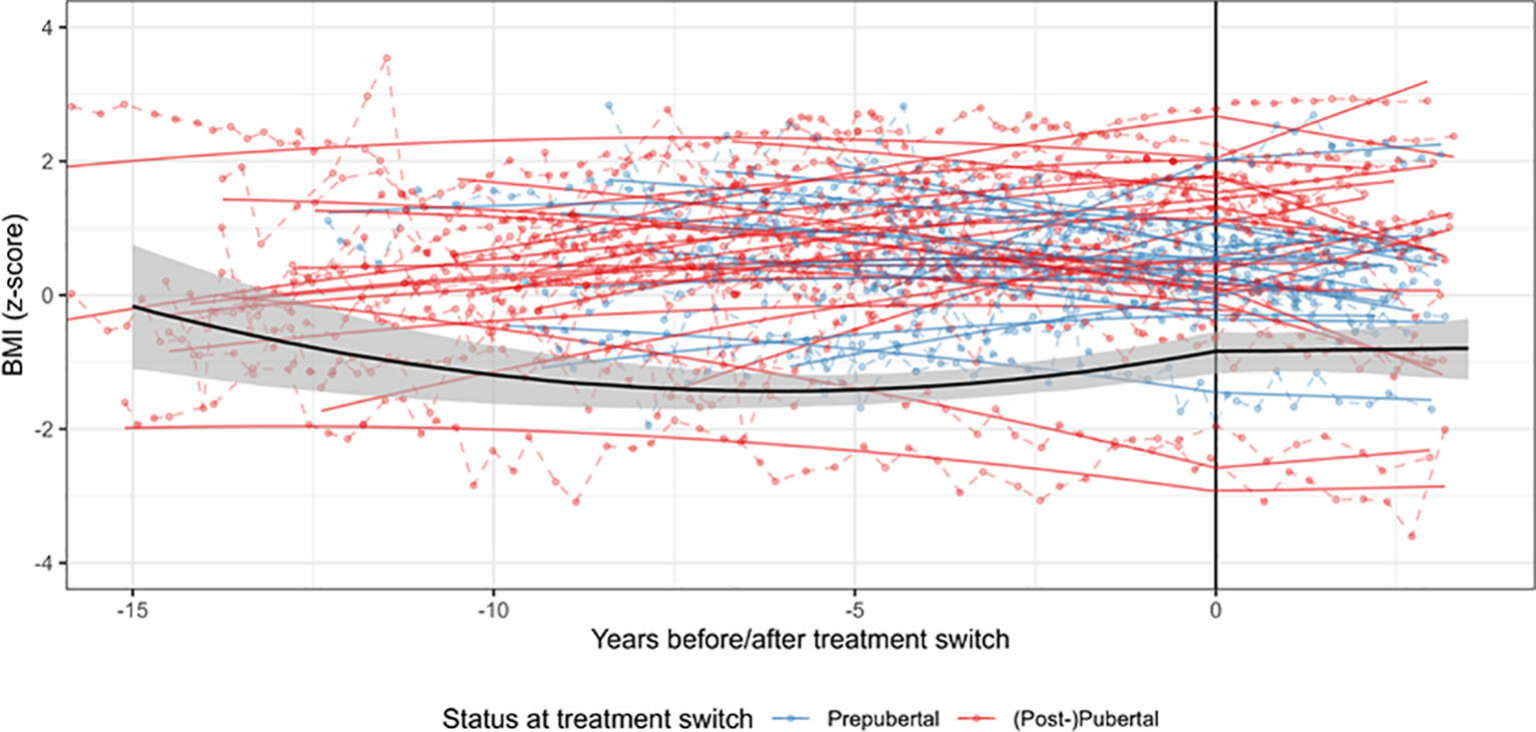
Colored observations and model fit of BMI z-scores. Observations are represented by dots and connected by dashed lines, model fit by patient (random effect) is represented by solid thin lines, and model fit across patients is represented by a black solid line with confidence bands in gray. Confidence bands are based on simulation and calculated as predictions over a reference grid. Marginal *R*² is 0.03, while conditional *R*² is 0.91. The model is fitted with fixed linear and quadratic time effect for the pre-period and a fixed linear interaction effect of time and period. Unstructured covariance matrix is specified with random linear time effect, random linear time–period interaction, and random intercept by patient, respectively. A total of 36 patients and 1,595 measurements were used in the model.

The analysis of body mass index (BMI) shows no shift of BMI change at treatment switch, with average slopes being close to horizontal post-switch and in the timespan 5 years prior to switch. Individual trajectories neither experience shifts in slopes after treatment switch. Children with BMI increasing over time before the switch maintained this trend after switch, while children with BMI decreasing over time pre-switch continued to show decreasing BMI values (**Figure 7**).

### Blood pressure

No effect of treatment change on sys-BP and dias-BP can be observed. The marginal average for sys-BP pre-switch is at -0.4 [-0.8, -0.1] *z*-scores per year and post-switch at -0.3 [-1.0, 0.4]. The marginal average level for dias-BP pre-switch is at -1.3 [-1.6, -1.1] *z*-scores per year and post-switch at -0.9 [-1.6, -0.2].

### HC dosing and FC dosing

**Figure 8:**
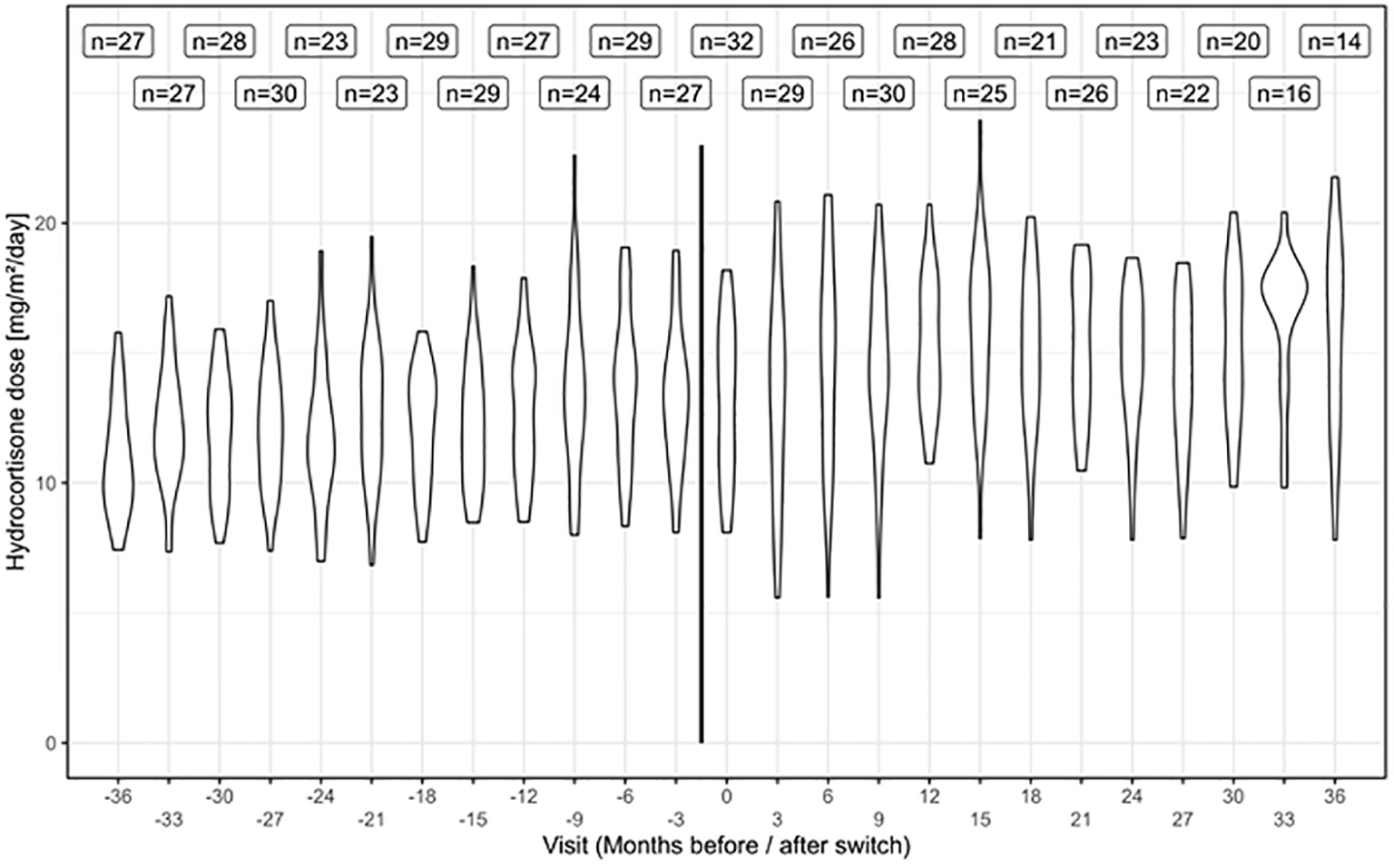
Density estimate of hydrocortisone dose at every visit. Treatment switch to HMRC is marked by a solid line, while visits to the left of the dotted line were before and visits to the right were after the treatment switch. The number of children included per visit is given above the respective density.

Average hydrocortisone dose was increased by 2.4 mg/sqm/day after switching to HMRC from 12.4 mg/m²/day before to a center of 14.8 mg/m²/day after treatment switch (**Table 2**, **Figure 8**). The sample size for subsequent visits is reduced because a significant proportion of patients have only recently switched and have not yet experienced visit 10 at month 27 after switch. The average fludrocortisone dose was increased by 11.7 µg/day during the treatment period with HMRC.

### Safety

No adrenal crisis was observed.

## Discussion

This is the first study reporting outcomes on the treatment of children and adolescents with 21-hydroxylase deficiency receiving a therapy with hydrocortisone modified-release capsules (Efmody^®^). Since its approval in 2021, 47 children and adolescents aged 4–25 years have been switched from immediate-release HC to HMRC therapy, while 36 were included in the assessment. The therapy switch to HMRC results in the reduction of morning 17-OHP levels into the range of acceptable disease control. The accelerated trend in growth rate was reduced after switching to HMRC therapy. In our study, especially in prepubertal children, growth channel approached the genetic parental target height percentile. However, the longest period of observation period is only 3.5 years. This trend is also evident in bone age. Here, too, there is a convergence between bone age and chronological age. There has been a decline in bone age acceleration.

In adults with CAH, HMRC has been reported to normalize 17OHP and androstenedione despite reduced hydrocortisone doses (7, 25). In our cohort, however, hydrocortisone dosage was slightly increased initially after the switch but remained within the recommended range, while dosage gradually decreased during follow-up. In the absence of clinical data, recommendations for treating children are derived from pharmacokinetic modeling (10), with two-thirds of the total dose administered at night and the remaining third in the morning (25). These data were used to grant authorization for use by people aged 12 years and over. Treating younger children would mean off-label treatment. In our cohort, HMRC treatment was initiated if the morning 17OHP levels were out of range or if a twice-daily dose was requested by the patient and parents, e.g., to avoid disturbing sleep at night or the necessity of HC dosing in the afternoon. Additionally, the children had to be treated with a single dose of at least 5 mg, and they had to be able to swallow capsules. Dose adjustment is also limited to 5-mg increments. The increased hydrocortisone dosage per square meter of body surface area after the switch may reflect the limited availability of HMRC capsule strengths of 5 and 10 mg, respectively. The approval of a 2.5-mg capsule would facilitate pediatric dosing, as all children, including those under 12 years, benefited from HMRC with improved morning 17OHP levels. Another reason for the slightly increased daily hydrocortisone dosage might be the hormonal phase of puberty, characterized by enhanced cortisol clearance resulting from reduced 11β-hydroxysteroid dehydrogenase type 1 activity (11β-HSD1), pubertal insulin resistance, and increased renal clearance, necessitating higher glucocorticoid doses (26).

Due to an assumed increased risk of glucocorticoid therapy for overweight or obesity starting already during childhood (27), these outcomes are relevant due to their contribution to cardiovascular risk. In our study, the treatment switch did not affect the BMI change during follow-up. One reason might be the slightly increased dosage of hydrocortisone after the switch to HMRC, possibly due to the lack of availability of dosages smaller than 5-mg capsules as discussed above.

In the prepubertal group in particular, the positive trend in growth rate leveled off. However, all treated children were closely monitored using 17OHP saliva profiles, which were requested regularly every 3 months and additionally within 2 to 3 weeks after a dose change of HC. No adrenal crisis was observed. In case of illness or fever, the families are instructed to continue treatment with HMRC at the usual dose and to administer an additional stress dose of immediate-release hydrocortisone.

It is recommended to take HMRC 1 h before or 2 h after a meal. This recommendation cannot always be strictly followed by children and adolescents, as they often go to bed closer to dinner time. The 17-OHP saliva test was carried out here according to the dosing schedule used. Subjectively, both parents and children reported increased satisfaction with the twice-daily dosing regime, especially the discontinued dosage at night and/or in the afternoon. This may partly explain the improved therapy monitoring, as adherence is higher with twice- than thrice-daily dosing. However, data on life satisfaction of children and parents were not collected.

In adult studies, reduced renin activity has also been observed with HMRC therapy, possibly due to the reduced formation of the MC receptor antagonist 17OHP (28). A reduction in fludrocortisone dose was not seen in our study group of growing patients.

Long-term studies are needed to further investigate the effectiveness of HRMC therapy in children and adolescents with regard to menarche and menstruation, onset of pregnancy in women, and semen quality in men. For this, it is necessary to conduct studies with larger numbers of cases and courses that last into adulthood. The I-SDM registry offers a suitable platform with a longitudinal module for the collection of follow-up data (https://sdmregistries.org/), which extends into adulthood and thus also covers aspects that are not yet primary for childhood, such as fertility (15).

### Limitations

Due to approval being granted in 2021, the duration of treatment observation with HMRC and the number of cases treated are still short, while statements on long-term returns are limited. In our study, subgroup analyses were not always possible and, when available, should be interpreted with caution. All reported *p*-values should be interpreted as exploratory. In blood pressure measurements, a large variance was indeed detectable, and large deviations for subsequent visits occur in our data. Due to possible measurement error, effects on blood pressure should be interpreted with caution. This is a pre–post-comparison study without a concurrent control group. Individuals are compared across time periods and serve as their own controls. The estimated effects may be biased if a patient’s time periods differ in aspects other than the administered medication—for example, for most patients, the COVID pandemic was present predominantly in the pre-switch period, which was shown to increase the rate of metabolically unhealthy children (29). Since *z*-scores of growth endpoints are used for analysis instead of raw data, measurement error is induced. The Robert-Koch Institute (RKI) suggests that absolute values >3 be interpreted with caution since in the reference cohort only few individuals with such extreme values are recorded. The visually assessed beneficial effect of treatment switch on alignment to parental target height is small and may be attributable to the phenomenon of regression to the mean. Due to the study design, no detailed analysis of adult height outcomes was planned. A control group could not be included, and the pre–post comparison may be subject to various sources of bias and confounding.

## Conclusion

Similar to the results observed in adults, switching to HMRC treatment resulted in reduced morning 17OHP levels in children and adolescents with CAH, suggesting an improved treatment control. Decreased body height growth rates and also of bone age were observed after the switch, and deviations from parental target height were reduced. Longer-term studies in larger cohorts extending into adulthood are needed to assess the adult height achieved and to draw conclusions about fertility and cardiovascular risk.

## Data Availability

The raw data supporting the conclusions of this article will be made available by the authors, without undue reservation.
